# Notices

**Published:** 1862-09

**Authors:** 


### NOTICES.

### LO! THE POOR SOLDIER!

The humanity of several ladies has prompted the organization of a society for the relief of Pennsylvania soldiers, in this city; and as that State is one of the largest patrons of our journal, it may answer a good purpose to say to them especially, that the contribution of a single half-dollar, in postage-stamps, will secure some item of material comfort—it may be to a neighbor, a friend, a cousin, a brother, a son. If you could know what warm thanks are sometimes bestowed upon the donors of a single orange or lemon, or a common cucumber-pickle, by men wasted with burning fever, or commencing scurvy; or what earnest expressions of gratitude have been made for a pair of darned old stockings, or holey cambric handkerchief, or old hat, or cane, by men who have reached here lame, without a hat, without a shoe, with nothing but shirt and ragged drawers. If these things could be known and witnessed by the notable housewives of the country, and by their warm-hearted daughters, known and witnessed as they really are every day in this city, we are sure that every home of every patriotic heart would be searched from garret to cellar for articles of clothing, a good deal the worse for wear, and which consequently could be spared, without a sacrifice worthy of mention, and thus an urgent good be done, without a felt cost. Let those who do not keep house send a few postage-stamps to "The Pennsylvania Relief Association, 176 Fulton Street, New-York;" or if by letter, address it to

Mrs. Dr. W. W. Hall,
Corresponding Secretary,
Care of Box 3349, New-York.

Among the first and most liberal of the benefactors of this Association are the wives of some of our wealthiest and most esteemed citizens. Mrs. William H. Aspinwall, by her contributions of money and clothing, and which is quite as valuable, by her warm, personal interest, and sincere words of approbation, encouragement, and good cheer, has shown herself a worthy wife of a worthy and high-minded and patriotic merchant, who, while other men have taken advantage of their country's emergencies, to make enormous charges for their services, and to swindle her in their contracts, has given the entire profits of his contracts, counting as nothing his time, and credit and risks amounting to tens of thousands, to the national treasury. Mrs. Robert Haydock, Mrs. General Wadsworth, and Alice Whitall, of Morristown, Pa., have been generous donors, while Mrs. Dr. Tyng, Mrs. Thomas I. Atwood, Mrs. Taturn and others, have not only given money and clothing, but large personal attention at the hospitals, in attending to the wants of the sick and suffering from their native State. Will not the daughters of Pennsylvania every where say: "Go on, sisters! and we'll help you"? Your Governor Curtin has found time to confer with the managers here in New-York personally, and has given the enterprise his countenance and his counsel; and with his accustomed self-denying and outshining patriotism and devotion, said to the ladies: "Secure your building, and I will pledge our State for the means of payment." The articles most imperatively wanted are good woolen shirts, woolen drawers, woolen socks, woolen wrappers, and woolen bandages; jars of pickles, cans of preserved meats, condensed milk, desicated vegetables, Boston crackers, papers of starch, farina, etc.

CURRENCY TABLE.

| | |
|---|---|
| One blue Franklin is worth, | 1 ct. |
| One yellow, " " | 30 cts. |
| One pink Washington, | 3 " |
| One green, " | 10 " |
| One black, " | 24 " |
| One blue, " | 30 " |
| One chocolate Jefferson, | 50 " |

Of the new stamp currency-notes there are four in number. The five and twenty cent stamps will be brown; the fifteen and twenty-five green.

PATRIOTS.—P. T. Barnum, with his usual liberality, has given five hundred dollars to the enlistment fund. Reader, go to "the Museum," next time you come to town. The beautiful aquaria are alone worth twenty-five cents to see.

GROVER AND BAKER, of sewing-machine notoriety, have given five thousand dollars. Remember that! you who are so far behind the times as to be yet without that most invaluable of all family-helps, a sewing-machine. But a higher honor is due to the inventor of all sewing-machines, and worthily the richest man in Connecticut, Elias Howe, Jr., who has not only given two thousand dollars for bounties, in addition to large sums before, but has become a private soldier himself, and thus has exhibited one of the very highest types of patriotism.

SOLDIER'S PAY.—A private enlisting under the new call for volunteers, if the war should close within twelve months, would receive, besides his regular rations and clothing, the following amount of money:

| | |
|---|---|
| State bounty, | $50 |
| Government advance bounty, | 27 |
| One month's advance pay, | 13 |
| Pay per year, | 156 |
| Government bounty at close of war, | 75 |
| Total, | $321 |
| Rations nine dollars per month—one year, | 108 |
| Clothing about, | 20 |
| Total one year's pay, | $449 |

This is exclusive of whatever bounty may be bestowed by individual patriotism; exclusive too of the proud consciousness of having periled health and limb and life itself in the hour of our country's trial. Let burning shame be to all laggards now.

SHOW YOUR COLORS NOW, by standing by the authority of the Government and eternal right. Let "precedent" be considered of secondary importance; let the Constitution itself, *as you interpret it merely*, be secondary. Whatever the authorities consider "constitutional" should not only be assented to by every good citizen, but should be sustained by his words and acts and influence. If he can not do these things conscientiously, let him be mute as a mouse, under the "violent presumption" that he is a fool, and has no better sense. For a sensible man will always diffidently mistrust the correctness of any opinion of his own, when he sees it is opposed to that of men of higher abilities.

"QUARE?"—What has all we have said about war to do with a "Journal of Health"? Suppose it has nothing to do with it, the Editor takes the bit in his mouth, and bids defiance to reins, "traces," shackles, harness and all. This is the time for acting up to the emergencies of the occasion, and to let all precedents go to the dogs, unless they demonstrably, at first glance, clearly contravene the self evident laws of human right and national existence. But this subject has something to do with health; for war maims its thousands; shatters for life the constitutions of thousands more; while the victims of camp diseases number tens of thousands, ay, scores of thousands; and we propose to *prevent* further calamities of this sort, by adopting the very first principles of all medical practice from the earliest ages down to the present hour; and that is, "*remove the cause*" of the disease first; until this is done, any attempt at a cure is utterly, absolutely, and always perfectly useless. The cause of the war, as all know, or ought to know, is "Slavery," as it exists among us. Remove it then to-morrow, by proclamation, and cause the reality to follow in due time, by keeping a million of conscripted, unmarried men, from twenty to thirty-five, in the field, *all the time;* as fast as a single man is rendered incapable from any cause, let his place be supplied from a reserved corps of conscripts, kept ready for marching orders at an hour's notice. Let this be the tune to which this war is set, and a million of men will be ready to march to its step in twenty days, and will sweep into the Gulf every enemy from the shores of the Atlantic to the great Pacific sea. When that is done, it will be plenty of time to inquire, "What shall be done next?" THE ANSWER TO ALL SUCH INQUIRIES SHOULD BE LEFT TO THE EXIGENCIES OF THE HOUR. Had this been done in the beginning, this war would have been ended triumphantly for human rights, and for the glory of our nation, before now. Let the creature do right always, and always will the benign Creator stand by him in the same, and will deliver, defend, and bless. This has always been the case with nationalities, and can never be otherwise, because "HE doeth his pleasure in the armies of heaven, and among the inhabitants of the earth," and "His pleasure" must always be the reward of right. Since the foregoing was sent to the printer, the President has called out six hundred thousand men in addition to those in the field. We wish every man from twenty to sixty had to draw, because most of the rich are beyond forty-five, and they are the very ones who could afford to pay a good price for able-bodied substitutes; these substitutes should be unmarried, between twenty-five and thirty, about five feet ten, and weighing not over one hundred and sixty pounds. Men of such a physique have strength and endurance. Boys under twenty-one are of very little account as soldiers; they soon get sick, and then some other soldier must take care of them.

BRAITHEWAITE'S RETROSPECT, for July, 1862—semi-annual, $1 25, two dollars a year, by W. A. Townsend, 39 Walker Street, New-York — Part 45, contains, as usual, the progress of medical science throughout the world for the preceding six months, in 152 different practical articles.

"SLEEP."—A second edition of our book on "Sleep," 360 pages, 12mo, $1.25, by mail, $1.40, is called for, and is now ready. It is the only book ever published for popular reading, which is truthful and wholly reliable as to a number of subjects connected with the hours of sleep. It is just such a book as every family ought to possess and ought to heed. If its suggestions were practically carried out, even as far as reasonably practicable without any expense of money, they would add to the lifetime both of parents and children, and would largely and infallibly promote the happiness, the moral purity, as well as the comfort, health and substantial thrift of every household.

In 1768, ninety-four years ago, the people of New-York had great faith in country air, for "Old Doctor Tillery took the boy (a sick son of Robert Bruce) up into the country." That is to "the house of entertainment, at the sign of the Black Horse," corner of Broadway and Barclay Street.

Exemption from Drafting.—All under eighteen, and all over forty-five years of age. All able-bodied men between those ages are liable to drafting; and they are "able-bodied," unless they have some physical disability. But what is physical disability? The Surgeon-general, Adjutant-general Hillhouse, in a recent order, declares that physical disability should in all cases be established to the satisfaction of the enrolling officer by a physician's certificate, as well as the affidavit of the party. He mentions the following imperfections as proper causes of disability: "Wounds of the head, which impair the faculties or cause convulsions; serious impairment of hearing, speech or vision; anchylosis, or active disease of any of the larger joints; the presence of pulmonary disease or organic disease of the heart; irreducible hernia; fistula in ano; large hemorrhoids; large and painful variscell or varicose veins which extend above the knee; the loss of a limb, or the thumb and forefinger on the right hand, or of any two fingers on either hand; the loss of the great toe; any marked physical imperfections which would unfit for active service."

Notice! Persons under eighteen and over forty-five are not *liable* for military duty or drafting. Such need not concern themselves about filing papers, getting certificates, etc.; they need just do nothing; for even if drafted, they would be at once relieved on proving their age. Hence such persons are not only "exempt," they are more, they are not "*liable.*" "Exempts" are able-bodied men between eighteen and forty-five, who from some physical infirmity, or from some office or calling, are excused. Even if a man is drafted, and draws a ticket to go to the war, he can hire a substitute, who must be a well, sound man, over twenty-one, and under forty-five. An exempt must be between eighteen and forty-five; the chief classes of exempts are "Shakers," Friends or Quakers, clergymen, school-teachers, mail stage-drivers or mail-carriers, telegraphic operators, engaged as such on or before the fourth of August, 1862, professors and students in all colleges or academies of learning, all persons honorably discharged from the army or navy of the United States, and all members and officers of Congress or State legislatures, or state or general government officers, or those engaged in the Post-office service.

